# Comparative Electrophysiological Analysis of Trigeminal and Dorsal Root Ganglion Neurons in Mice

**DOI:** 10.1523/ENEURO.0269-26.2026

**Published:** 2026-09-18

**Authors:** Sachin Goyal, Nesia A. Zurek, Sascha R. A. Alles

**Affiliations:** Department of Anesthesia, Cincinnati Children’s Hospital Medical Center, Cincinnati, Ohio 45229

**Keywords:** dorsal root ganglia, electrophysiology, sensory neurons, trigeminal ganglia

## Abstract

Cultured dissociated trigeminal ganglion (TG) and dorsal root ganglion (DRG) neurons are widely used to study peripheral sensory function, yet direct electrophysiological comparisons under identical experimental conditions remain limited. We compared intrinsic electrophysiological properties of mouse TG (mTG) and mouse DRG (mDRG) neurons using whole-cell patch-clamp electrophysiology from male mice. In addition to conventional comparisons of membrane properties, action potential waveform characteristics, and firing behavior, neurons were stratified by soma size and analyzed using principal component analysis (PCA) and Pearson’s correlation analyses to determine ganglia-specific electrophysiological signatures. mTG neurons exhibited enhanced stimulus-evoked excitability compared with mDRG neurons, characterized by shorter first spike latency, increased repetitive firing, and greater action potential output despite similar resting membrane potential, rheobase, and input resistance. These differences were primarily driven by small-diameter neurons, which displayed increased rebound and repetitive firing, whereas differences in spontaneous activity were predominantly observed in large-diameter neurons. PCA revealed distinct clustering of TG and DRG neurons based on electrophysiological properties, while Pearson’s correlation analyses demonstrated tissue-specific relationships among electrophysiological parameters, particularly involving afterhyperpolarization, indicating that coordinated regulation of excitability differs between sensory ganglia. These findings demonstrate that TG and DRG neurons differ not only in individual electrophysiological properties but also in the coordinated organization of those properties. Together, these data provide a functional framework for understanding ganglion-specific regulation of peripheral sensory neuron excitability and establish a foundation for future mechanistic studies and the development of targeted therapies for peripheral pain disorders.

## Significance Statement

Trigeminal ganglion (TG) and dorsal root ganglion (DRG) neurons are fundamental models for studying peripheral sensory physiology and pain mechanisms. Although previous studies have identified differences in their anatomy, central circuitry, and transcriptomic profiles, direct comparisons of their intrinsic electrophysiological properties under identical experimental conditions have been lacking. Here, we demonstrate that cultured dissociated TG and DRG neurons exhibit distinct excitability profiles, firing behaviors, and coordinated electrophysiological signatures. These findings establish a functional framework linking molecular diversity to neuronal function, provide an important reference for interpreting studies of peripheral sensory neurons, and may facilitate the identification of ganglion-specific therapeutic strategies for sensory diseases such as neuropathic pain.

## Introduction

Vertebrates detect a broad range of external and internal stimuli through functionally distinct peripheral sensory neurons whose cell bodies are located within the trigeminal ganglia (TG) and dorsal root ganglia (DRG; Jones et al., 2026). TG and DRG neurons encode somatosensory modalities including nociception, touch, temperature, and proprioception but innervate distinct anatomical regions. TG neurons relay sensory information from the head and face through the trigeminal nucleus caudalis, whereas DRG neurons transmit sensory information from the limbs, trunk, and viscera through the dorsal horn of the spinal cord (Colloca et al., 2017; Rodriguez et al., 2017; Megat et al., 2019; Messlinger et al., 2020; Su et al., 2026). Nociceptors within both ganglia detect noxious stimuli, and their electrophysiological properties contribute to the processing of acute and chronic pain.

Despite their shared sensory functions, TG and DRG neurons exhibit important biological differences. Clinically, TG dysfunction contributes to craniofacial pain disorders including migraine and trigeminal neuralgia, whereas DRG dysfunction underlies peripheral neuropathic pain conditions such as radiculopathy, diabetic neuropathy, and chemotherapy-induced peripheral neuropathy (Basbaum et al., 2009; Colloca et al., 2017; Messlinger et al., 2020). These ganglia also differ in embryonic origin, transcriptional programs, receptor expression, neuronal subtype composition, and responses to injury (Zou et al., 2004; Price and Flores, 2007; Durham and Garrett, 2010; Usoskin et al., 2015; Messlinger et al., 2020). For example, peripheral nerve injury induces sympathetic sprouting in the DRG but not the TG (Bongenhielm et al., 1999; Chien et al., 2005; Xie et al., 2007). Together, these observations suggest that TG and DRG neurons possess distinct molecular and functional properties that may influence peripheral pain mechanisms.

Cultured dissociated mouse trigeminal ganglion neurons (mTG-Ns) and mouse dorsal root ganglion neurons (mDRG-Ns) are widely used to study peripheral somatosensation including nociception. Historically, peripheral sensory neuron subtypes have been classified based on cell size, degree of myelination, and nerve conduction velocity. In mouse tissue, large-diameter (>25 µm), myelinated, fast-conducting neurons (2–55 m/s) are classified as A-fibers, whereas small-diameter (<25 µm), unmyelinated, slow-conducting neurons (<2 m/s) are classified as C-fibers (Bhuiyan et al., 2024; Jones et al., 2026). For drug discovery, compound screening, and target validation studies, the functional properties of mTG-Ns and mDRG-Ns are commonly evaluated using patch-clamp electrophysiology, a gold standard method for characterizing neuronal excitability and ion channel activity (Neher and Sakmann, 1976; Harrison et al., 2015; Ghovanloo et al., 2025). Although TG and DRG neurons share many sensory functions, differences in their genetic and molecular signatures suggest the existence of corresponding functional differences at the electrophysiological level (Su et al., 2026). Therefore, the objective of this study was to systematically compare the electrophysiological properties of mTG-Ns and mDRG-Ns.

We characterized intrinsic electrophysiological properties, including rheobase, resting membrane potential (RMP), input resistance, first spike latency (FSL), and action potential (AP) waveform properties, in 54 mTG-Ns (14 mice) and 65 mDRG-Ns (15 mice). Furthermore, we evaluated neuronal subtypes from mice based on cell size (small and large) to determine whether size-dependent differences contribute to the functional heterogeneity observed between these sensory ganglia. To further define functional differences between sensory ganglia, we complemented traditional electrophysiological comparisons with principal component analysis (PCA) and correlation-based analyses to examine coordinated relationships among electrophysiological features at both the tissue- and size-specific levels. Our analysis identified several significant electrophysiological differences between mTG-Ns and mDRG-Ns, indicating that these sensory neuron populations possess distinct functional properties. These findings advance our understanding of peripheral sensory neuron diversity and provide important context for the use of mouse TG and DRG in studies of pain mechanisms, pharmacological screening, and therapeutic target validation.

## Materials and Methods

### Animals

All animal research was conducted with the approval of the Cincinnati Children's Hospital Medical Center and University of New Mexico Institutional Animal Care and Use Committee. Male BALB/cJ mice (20–25 g: 5–6 weeks old) were obtained from Jackson Laboratories. Rodents were housed in a well-ventilated room maintained at 20–22°C. They were acclimated for 1 week prior to the commencement of the studies and had unrestricted access to food and water throughout the experimental period.

### Cell culture

#### Trigeminal ganglion culture

Mouse TG cultures were performed as previously described by our group (Goyal et al., 2025a). In brief, mice were put under deep anesthesia using 3% isoflurane for 2–3 min and then decapitated using scissors for dissection of the trigeminal ganglion (TG) following the opening of the skull and extraction of the brain to reveal the trigeminal nerve ganglia. The TGs were extracted from the connective tissue and dura mater at the cranium's base. The ganglia were put in a 5 cm culture dish containing ice-cold Hanks balanced salt solution (HBSS) without Ca^2+^/Mg^2+^ (STEMCELL Technologies, catalog #37250). Using a scalpel, the ganglia were mechanically disrupted and cut into smaller fragments. The tissue was transferred to a 1.5 ml tube prefilled with ice-cold HBSS, and then spun down for 1 min at 300 × *g* to form a pellet. The supernatant was aspirated, and the ganglia underwent a 20 min digestion period at 37°C in an enzymatic solution composed of HBSS, papain (1 mg protein/ml, Worthington Biochemical, catalog #LS003126), and ʟ-cysteine (5.5 mM, Sigma, catalog #C7352-25g) and were gently agitated at 10 min. The papain-digested tissue suspension was spun down for 1 min at 200 × *g*, the supernatant removed, and a digestion solution added which is composed of HBSS, collagenase type 2 (6 mg/ml, Worthington Biochemical, catalog #LS004176), and dispase II (4 mg/ml, Sigma, catalog #D4693). Digestion was carried out for 20 min at 37°C and gently agitated at 10 min. The digested tissue suspension was spun down for 4 min at 400 × *g*. The supernatant was removed and resuspended in 0.5 ml warm complete Leibovitz's L-15 medium containing L-15 (Quality Biological, catalog #112-029-101) with 5% fetal bovine serum (Invitrogen, catalog #26140-079), 2% 1 M HEPES (Sigma, catalog #H0887), and 1% Penicillin-Streptomycin (Invitrogen, catalog #15070-063). The suspension was triturated 10 times with a 200 µl pipette tip to ensure that the tissue was well digested, and the suspension became uniformly cloudy. Next, the digested tissue suspension was carefully placed on top of a Percoll gradient [12.5% Percoll (Sigma, catalog #P4937) layered on top of 28% Percoll] and spun at 1,300 × *g* for 10 min. Taking care not to disturb the lower layers of the spun down gradient, 4.5 ml of supernatant was aspirated from the top to remove most of the debris. A 4.5 ml warm complete L-15 medium was added to the remaining solution in the tube. Centrifugation was conducted again for a duration of 6 min at a speed of 1,000 × *g*. Once the supernatant was removed, the pellet was resuspended in 0.5 ml of warm complete Dulbecco's modified Eagle medium (DMEM; Invitrogen, catalog #11995-065) containing 10% fetal bovine serum and 2% Penicillin-Streptomycin. Then, 125 µl of the mouse TG cell suspension was added to each 12 mm coverslip, precoated with poly-d-lysine (20 µg/ml, Invitrogen, catalog #A38904-01), and coated with laminin (16 µg/ml, Sigma, catalog #L2020). It was allowed to attach for 40–60 min before gently flooding the wells with enough complete DMEM media to fill each well, 1–2 ml for a 12-well plate. Following cell plating, cells were incubated at 37°C and 5% CO_2_ until they were used.

#### Dorsal root ganglion culture

Mouse DRG cultures were prepared as previously described by our group (Goyal et al., 2025a,b). In brief, mice were deeply anesthetized with 3% isoflurane and then killed by decapitation prior to dissecting the DRG for primary cultures intended for electrophysiological study. Bilateral L3-L5 DRGs were carefully removed and placed in ice-cold HBSS without Ca^2+^/Mg^2+^ (STEMCELL Technologies, catalog #37250). Using a scalpel, the ganglia were mechanically disrupted and cut into smaller fragments. Subsequently, the ganglia underwent a 20 min digestion period in an enzymatic solution composed of HBSS, papain (1 mg protein/ml, Worthington Biochemical, catalog #LS003126), and ʟ-cysteine (5.5 mM, Sigma, catalog #C7352-25g). This was followed by another 20 min digestion in a solution containing HBSS, dispase II (4 mg/ml, Sigma, catalog #D4693-1g), and collagenase type 2 (6 mg/ml, Worthington Biochemical, catalog #LS004176). Both enzymatic digestions were carried out at 37°C, gently agitated every 10 min. The digested ganglia were again suspended in 10% fetal bovine serum (Invitrogen, catalog #26140-079) and 2% Penicillin-Streptomycin (Invitrogen, catalog #15070-063) in DMEM (Invitrogen, catalog #11995-065). Pasteur pipettes that had been flame polished were used to triturate the suspension until it was readily able to flow through the hole. Cells were plated on 12 mm glass coverslips and coated with 20 µg/ml poly-d-lysine (Invitrogen, catalog #A38904-01) and 16 µg/ml laminin (Sigma, catalog #L2020) in HBSS without Ca^2+^/Mg^2+^. Following cell plating, cells were incubated at 37°C and 5% CO_2_ until they were used.

### Whole-cell patch-clamp electrophysiology

Whole-cell patch-clamp electrophysiology was done as previously described by our group (Zurek et al., 2024a; Goyal et al., 2025a,b). Neurons were identified using differential interference contrast optics linked to an IR-2000 digital camera (Dage-MTI). The size was determined by utilizing Dage-MTI camera software or ImageJ (NIH) calibrated using microscale calibration slide. Current-clamp recordings were done with a MultiClamp 700B (Molecular Devices). Signals were acquired using a Digidata 1550B converter (Molecular Devices) and recorded using Clampex 11 software (Molecular Devices). Patch pipettes were prepared using a Zeitz puller (Werner Zeitz) from borosilicate thick glass (GC150F, Sutter Instrument). Bridge equilibrium was used in all the recordings. Cell capacitance was calculated using the whole-cell capacitance compensation circuit in MultiClamp 700B (Molecular Devices). Cells that did not fire APs or had an RMP depolarized to at least −35 mV or had an access resistance exceeding 15 MΩ were excluded from further analysis.

For TG and DRG neuron recordings, the internal solution comprised the following (in mM): 125 K-gluconate, 6 KCl, 10 HEPES, 10 EGTA, 2 Mg-ATP, with pH adjusted to 7.3 using KOH and an osmolarity range of 290–310 mOsm. The resistance of the electrode was between 4 and 8 MΩ. Electrophysiological measurements were carried out between 16 and 30 h after cells were placed in the culture dish. Junction potential was not adjusted for in the recordings. While recording, the cultured cells were continuously perfused with warm and carbogen-bubbled artificial cerebrospinal fluid (aCSF) containing the following (in mM): 113 NaCl, 3 KCl, 25 NaHCO_3_, NaH_2_PO_4_, 2 MgCl_2_, 2 CaCl_2_, and 11 dextrose, pH 7.4.

### Electrophysiology analysis methods

Cell diameter was used to sort the cells by size; small cells had a diameter <25 µm, and large cells were ≥25 µm. Current-clamp step recordings began with a 25 ms baseline period during which neurons remained at rest, followed by a 500 ms current injection protocol. Current steps were applied in 10 pA increments, starting at −100 pA and increasing progressively until the neuron reached firing inactivation or until a maximum current of 2 nA was delivered. Each current step was separated by a 500 ms recovery period. Electrophysiological analyses were performed using Easy Electrophysiology software as previously described by our group (Zurek et al., 2024a,b; Goyal et al., 2025a,b). Rheobase was defined as the smallest depolarizing current injection that elicited at least one AP, excluding spontaneous activity and rebound firing following hyperpolarization. Because current injections increased in 10 pA increments, the minimum measurable rheobase was 10 pA. Neurons were stimulated sequentially until rheobase was reached and then further depolarized until firing inactivation occurred, defined as the point at which AP generation ceased or the number of APs declined after reaching a maximal firing rate. RMP was determined by averaging at least 10 baseline sweeps and was not corrected for the liquid junction potential. Input resistance (*R*_in_) was calculated from the membrane voltage response to a hyperpolarizing −100 pA current step. FSL was measured using Easy Electrophysiology software as the time interval between the onset of current injection and the peak of the first AP. The frequency–current (*F*–*I*) relationships were generated using only neurons that exhibited repetitive firing. For these analyses, the number of APs generated at each depolarizing current step above rheobase was quantified and analyzed using GraphPad Prism version 10.0.2. The maximum number of APs (Max APs) was defined as the greatest number of APs produced by a neuron during any individual current injection step. Rebound firing was defined as AP firing during the 500 ms recovery in any of the hyperpolarizing current injection steps. Cells that fired more than one AP during any current injection step at rheobase or higher were classified as multifiring. APs from spontaneous activity were excluded in determining multifiring cells.

Spontaneous activity was assessed with either no current injection or injecting enough current injection to hold the membrane voltage at −45 mV during a 30 s recording. Neurons that had one or more APs during the 30 s were classified as having spontaneous activity. Rebound firing was defined as the occurrence of one or more APs during the 500 ms recovery period following any hyperpolarizing current injection step. Spontaneous activity was assessed under either resting conditions (0 pA current injection) or by injecting sufficient current to maintain the membrane potential at approximately −45 mV during a 30 s recording period. Neurons that generated one or more APs during this interval were classified as exhibiting spontaneous activity.

AP waveform and phase plot analyses were performed using the AP elicited at rheobase. Waveform and phase plot parameters were generated using the AP Kinetics Analysis module in Easy Electrophysiology software. The AP peak was defined as the maximum membrane potential reached during the AP. AP amplitude was calculated as the difference between the AP threshold and the AP peak. Threshold detection was performed using the method II (Sekerli et al., 2004), with analysis constrained to a window extending up to 4 ms before the AP peak; all threshold determinations were subsequently verified by visual inspection. The afterhyperpolarization (fAHP) amplitude was defined as the difference between the AP threshold and the most negative membrane potential reached during the hyperpolarization phase following the AP. AP rise time and decay time were calculated using 10–90% amplitude cutoffs. Maximum rise slope is defined as the maximum first derivative of membrane voltage with respect to time (dV/dtmax) during the depolarization phase of the AP, while the maximum decay slope is the most negative first derivative of membrane voltage (dV/dt) during the repolarization phase of the action potential. AP half-width was calculated automatically by Easy Electrophysiology using these predefined parameters. Phase plots were generated in Easy Electrophysiology by calculating the first derivative of membrane voltage (dV/dt) over time and plotting dV/dt against membrane potential. For each AP, data were analyzed within a time window extending 20 ms before the AP peak and 50 ms after the AP peak to capture the complete AP waveform. Phase plots were subsequently examined to assess the presence of an AP shoulder, which was identified from characteristic features in the repolarization phase of the curve, as previously described (Davidson et al., 2014).

To assess neuronal excitability during gradually increasing depolarization, neurons were subjected to a 1 s linear current ramp from 0 to 2 nA. Each ramp was followed by a 1 s recovery period before being repeated for a total of three sweeps. Unless otherwise indicated, analyses were performed using the first ramp sweep to minimize potential effects of activity-dependent changes in excitability across repeated stimulations. Neurons were classified as either ramp responsive or nonresponsive based on the presence or absence of action potential firing during the ramp. For ramp-responsive neurons, the total number of action potentials (Ramp Spike Number), first spike latency (Ramp FSL), and interspike interval (Ramp ISI) were quantified using Easy Electrophysiology. Ramp FSL was defined as the time from the onset of the current ramp to the first evoked action potential, and Ramp ISI was calculated as the mean interspike interval between consecutive action potentials during the ramp.

### Principal component analysis

Electrophysiological features were averaged at the level of each mouse within tissue and size group prior to multivariate analysis. Specifically, neurons were separated into TG and DRG populations and further classified as small or large based on soma size, generating four groups: TG small, TG large, DRG small, and DRG large. A mouse was included in a given tissue and size group only if at least three neurons of that size were successfully recorded for that mouse. For each mouse meeting this inclusion criterion within a given group, mean values were calculated for electrophysiological properties including intrinsic membrane properties, action potential waveform features, and ramp-evoked firing parameters. Features included RMP, *R*_in_, rebound prevalence, rheobase, FSL, proportion of multifiring neurons, maximum number of action potentials, action potential peak, amplitude, threshold, rise time, decay time, half-width, afterhyperpolarization (fAHP), and ramp-evoked measures [Ramp prevalence (Ramp), spike number (Ramp_Spike), interspike interval (Ramp_ISI), and first spike latency (Ramp_FSL)].

PCA was performed in Python using JupyterLab and scikit-learn on the mouse-averaged datasets (McKinney, 2010; Pedregosa et al., 2011; Kluyver et al., 2016). Prior to PCA, all electrophysiological features were converted to numeric values, features containing only missing values were excluded, and features with zero variance across samples were removed. The remaining missing values were imputed using the mean value of that feature across samples. To account for differences in scale among electrophysiological parameters, each feature was standardized by *z*-scoring prior to PCA. PCA was then performed on the standardized feature matrix, and the first principal components were used to visualize separation among TG and DRG populations by size. The proportion of variance explained by each principal component was calculated from the PCA model. Feature loadings for each principal component were extracted from the component loading matrix to identify the electrophysiological parameters contributing most strongly to separation across samples.

To further visualize similarity among donor averages, pairwise Euclidean distances were calculated between samples in PCA space using the first two principal components (PC1 and PC2) and displayed as a heatmap ordered by tissue and size group.

### Correlation analysis

Pearson’s correlation coefficients were calculated independently for TG and DRG neurons using Python in JupyterLab. Correlation analyses were performed on all neurons combined as well as separately for small and large neurons. Neurons were classified as small (<25 µm soma diameter) or large (≥25 µm soma diameter). Electrophysiological features included RMP, *R*_in_, rebound prevalence, rheobase, FSL, proportion of multifiring neurons, maximum number of action potentials, action potential peak, amplitude, threshold, rise time, decay time, half-width, fast afterhyperpolarization (fAHP), and ramp-evoked parameters [Ramp prevalence (Ramp), spike number (Ramp_Spike), interspike interval (Ramp_ISI), and first spike latency (Ramp_FSL)].

Pearson’s correlation matrices were generated independently for TG and DRG populations using pairwise complete observations. To identify tissue-specific differences in the relationships among electrophysiological features, a difference correlation matrix (Δ*r*) was calculated by subtracting the DRG correlation matrix from the corresponding TG correlation matrix (Δ*r* = rTG − rDRG). Positive values indicate that the correlation coefficient was more positive in TG than in DRG, whereas negative values indicate that the correlation coefficient was more negative in TG than in DRG. Correlation matrices were visualized as lower triangular heatmaps to remove redundant information arising from matrix symmetry. Custom analysis and visualization scripts were written in Python using the pandas, NumPy, Matplotlib, and Seaborn libraries and will be made available upon publication.

### Statistical analyses

Easy Electrophysiology v.2.5.1 and Clampfit 11.2 (Molecular Devices) were used for conducting the electrophysiological analysis. Statistical analysis was conducted with GraphPad Prism v10.0.2. Error bars denote mean ± standard error of the mean (SEM) unless otherwise specified. A *p* value of <0.05 was considered statistically significant. Statistical tests are shown in the figure legends.

### Code availability

Custom Python scripts/Jupyter notebooks used for PCA, pairwise distance analysis, and data visualization are available at https://github.com/AllesLab/Goyal_DRG_TG.

## Results

**Figure 1.**
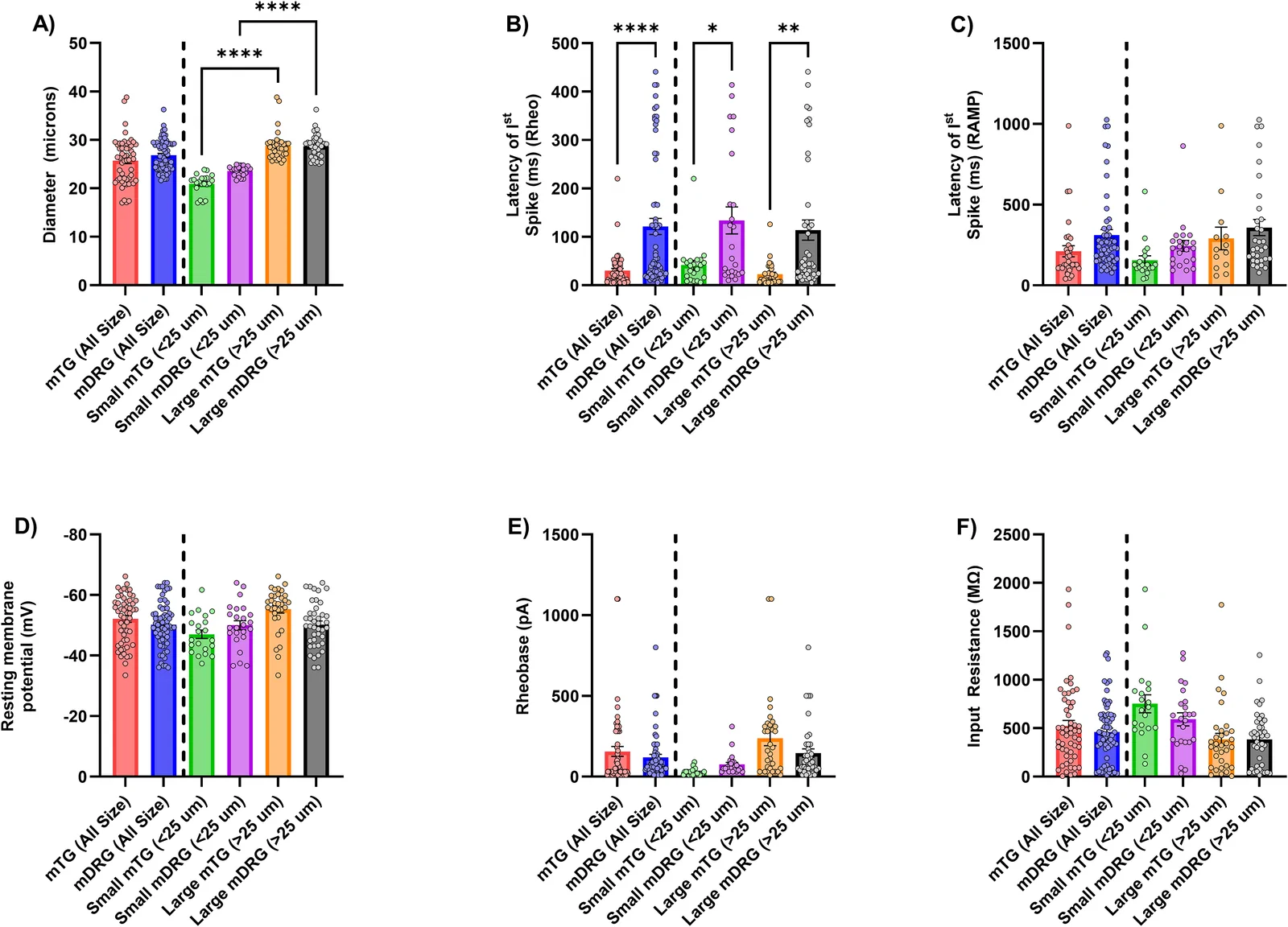
Intrinsic electrophysiological differences were observed between mTG cells (*n* = 54) and mDRG cells (*n* = 65) during current-clamp recording. ***A***, Cell diameter was measured during patch-clamp recording with an IR-2000. ***B***, Significant difference was found in latency of first spike at rheobase (mTG <25 µm *n* = 21, >25 µm *n* = 33 and mDRG <25 µm *n* = 24, >25 µm *n* = 41, ****p < 0.0001, **p < 0.01, *p < 0.05; ordinary one-way ANOVA followed by Tukey's multiple comparison). ***C***, Latency of first spike during 500 pA RAMP protocol (mTG *n* = 32, mDRG *n* = 54, mTG <25 µm *n* = 19, >25 µm *n* = 13 and mDRG <25 µm *n* = 22, >25 µm *n* = 32); ***D***, RMP; ***E***, rheobase; and ***F***, input resistance were not significantly changed. Error bars represent mean and SEM. ANOVA, analysis of variance.

Distinct electrophysiological differences were observed between mTG and mDRG neurons across multiple properties. In addition to comparing electrophysiological characteristics between the two neuronal populations, differences based on cell size were also examined within each group. Neurons from both mTG and mDRG were classified according to cell diameter as small (<25 µm) or large (>25 µm). Cells were distributed similarly across size categories in both groups, with mTG consisting of 38% small and 62% large neurons and mDRG consisting of 37% small and 63% large neurons. Neuronal diameters ranged from 17 to 38 µm. A significant size difference was observed between small- and large-diameter mTG neurons (20.95 ± 0.45 µm vs 28.76 ± 0.53 µm, respectively; *p* < 0.0001). Similarly, small mDRG neurons displayed a significant size difference than large mDRG neurons (23.59 ± 0.20 µm vs 28.70 ± 0.36 µm, respectively; *p* < 0.0001). Electrophysiological comparisons were subsequently performed between small mTG neurons (20.95 ± 0.45 µm) and small mDRG neurons (23.59 ± 0.20 µm), which showed no significant difference in diameter (*p* = 0.0742). Similarly, comparisons between large mTG neurons (28.76 ± 0.53 µm) and large mDRG neurons (28.70 ± 0.36 µm) revealed no significant difference in diameter (*p* > 0.9999; Fig. 1*A*).

### Mouse TG neurons fired earlier in response to current injection than mouse DRG neurons

Intrinsic membrane properties were analyzed in all recorded neurons. Cell diameter did not differ significantly between mTG and mDRG neurons (25.72 ± 0.63 µm vs 26.81 ± 0.39 µm, respectively; *p* = 0.4523; Fig. 1*A*). In contrast, the FSL evoked by stepwise current injection differed significantly between the two neuronal populations. mTG neurons fired earlier following current injection (30.19 ± 4.25 ms), whereas mDRG neurons exhibited a substantially longer latency to first action potential generation (121.31 ± 16.69 ms; *p* < 0.0001; Fig. 1*B*). However, no significant difference in FSL evoked by a 500 pA ramp protocol (Fig. 1*C*). When analyzed by cell size, significant differences in FSL at rheobase were also evident. Small mTG neurons exhibited a shorter FSL than small mDRG neurons (41.67 ± 5.54 ms vs 133.92 ± 17.05 ms, respectively; *p* = 0.0336). Similarly, large mTG neurons displayed a significantly shorter FSL than large mDRG neurons (22.89 ± 2.93 ms vs 113.93 ± 16.63 ms, respectively; *p* = 0.0025; Fig. 1*B*). The RMP was not significantly different between mTG and mDRG neurons (−52.07 ± 1.08 mV vs −50.17 ± 0.92 mV, respectively; *p* = 0.7378; Fig. 1*D*). Likewise, rheobase did not differ significantly between mTG and mDRG neurons (156.11 ± 31.07 pA vs 119.0 ± 17.66 pA, respectively; *p* = 0.8825; Fig. 1*E*). *R*_in_ was also comparable between the two groups (522.93 ± 56.80 MΩ for mTG vs 461.55 ± 39.16 MΩ for mDRG; *p* = 0.9353; Fig. 1*F*). No significant size-dependent differences between mTG and mDRG neurons were observed for FSL (ramp-evoked), RMP, rheobase, or *R*_in_ (Fig. 1*C*–*F*).

### Mouse TG neurons displayed greater excitability than mouse DRG neurons

**Figure 2.**
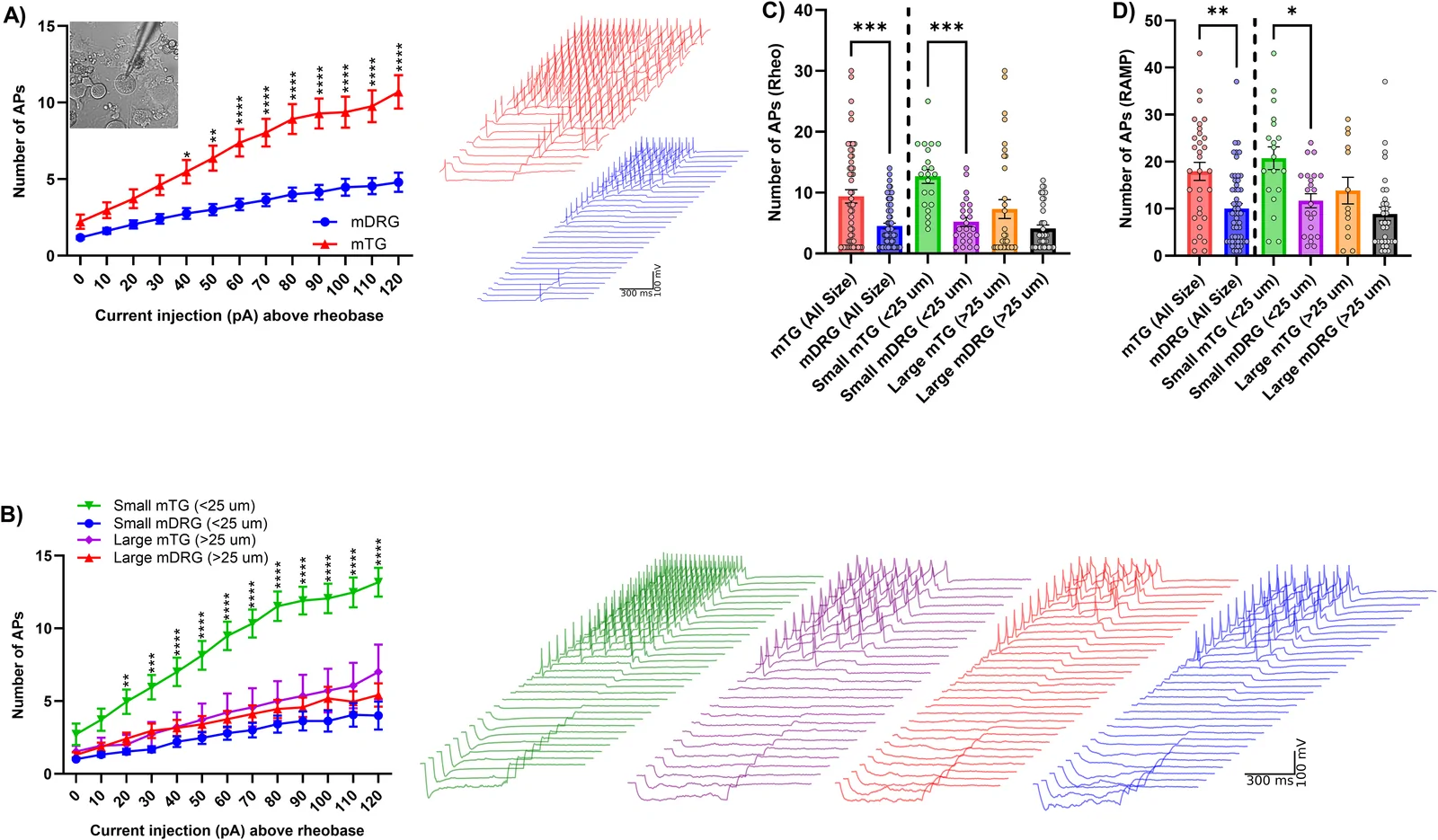
Frequency measured as a function of injected current level above rheobase. ***A***, ***B***, A significant difference was found in the firing frequency (restricted to multifiring cells) during the current step protocol (10 pA stepwise increase of 500 ms current injection window) as current injection increased from rheobase (mTG *n* = 36 and mDRG *n* = 45 cells, ****p < 0.0001, **p < 0.01, *p < 0.05; two-way ANOVA followed by Sidak's multiple comparison). Inset, Representative traces. Red, mTG and blue, mDRG examples of firing at −100 pA, rheobase, and multifiring during a 500 ms current injection step. mTG <25 µm *n* = 21 and >25 µm *n* = 15 versus mDRG <25 µm *n* = 19 and >25 µm *n* = 26 cells, respectively. Two-way ANOVA followed by Tukey's multiple comparison. Inset, Representative traces. Green, mTG (<25 µm); purple, mTG (>25 µm); red, mDRG (>25 µm); and blue, mDRG (<25 µm). Significant difference was observed in ***C***, number of action potential elicited on rheobase (mTG *n* = 54, mDRG *n* = 65) and ***D***, during 500 pA RAMP protocol (mTG *n* = 32, mDRG *n* = 54) between mTG cells and mDRG cells during current-clamp recording (***p < 0.001, **p < 0.01, *p < 0.05; ordinary one-way ANOVA followed by Tukey's multiple comparison). Error bars represent mean and SEM. ANOVA, analysis of variance.

Neuronal excitability in mTG and mDRG neurons was assessed using stepwise current injections extending to at least 120 pA above rheobase. Only APs evoked during the 500 ms current injection were included in the analysis; rebound and spontaneous APs were excluded. Compared with mDRG neurons, mTG neurons generated significantly more APs at suprathreshold current injections up to at least 120 pA above rheobase (****p < 0.0001, **p = 0.0013, *p = 0.0202; two-way ANOVA followed by Sidak's multiple-comparison test; Fig. 2*A*). Analysis of the frequency–current (F–I) relationship revealed a significant size-dependent effect, with small mTG neurons firing more APs than small mDRG neurons (****p < 0.0001, ***p = 0.0006, **p = 0.0098; two-way ANOVA followed by Tukey's multiple-comparison test; Fig. 2*B*). To further quantify excitability, the maximum number of APs generated by each neuron at any current level above rheobase was determined, including both single- and multifiring cells. mTG neurons exhibited a significantly greater maximum AP output than mDRG neurons (9.38 ± 1.10 vs 4.52 ± 0.45 APs, respectively; *p* = 0.0002; Fig. 2*C*). This difference was primarily driven by small-diameter neurons, as small mTG neurons generated significantly more maximum APs than small mDRG neurons (12.66 ± 1.15 vs 5.20 ± 0.75 APs, respectively; *p* = 0.0006; Fig. 2*C*). Similar results were observed during the 500 pA ramp protocol. mTG neurons fired significantly more APs than mDRG neurons (17.93 ± 1.47 vs 10.03 ± 0.95 APs, respectively; *p* = 0.0014; Fig. 2*D*). Again, this effect was most pronounced in small-diameter neurons, with small mTG neurons exhibiting greater maximum AP firing than small mDRG neurons (20.73 ± 2.30 vs 11.68 ± 1.42 APs, respectively; *p* = 0.0171; Fig. 2*D*). Collectively, these findings demonstrate that mTG neurons are intrinsically more excitable than mDRG neurons, exhibiting enhanced repetitive firing in response to stimulation. The greater excitability is largely attributable to the small-diameter mTG neuronal population, which displays substantially higher firing frequencies and maximum AP output than corresponding mDRG neurons.

### Electrophysiological differences were observed in phenotypic firing patterns between mouse TG and DRG neurons

**Figure 3.**
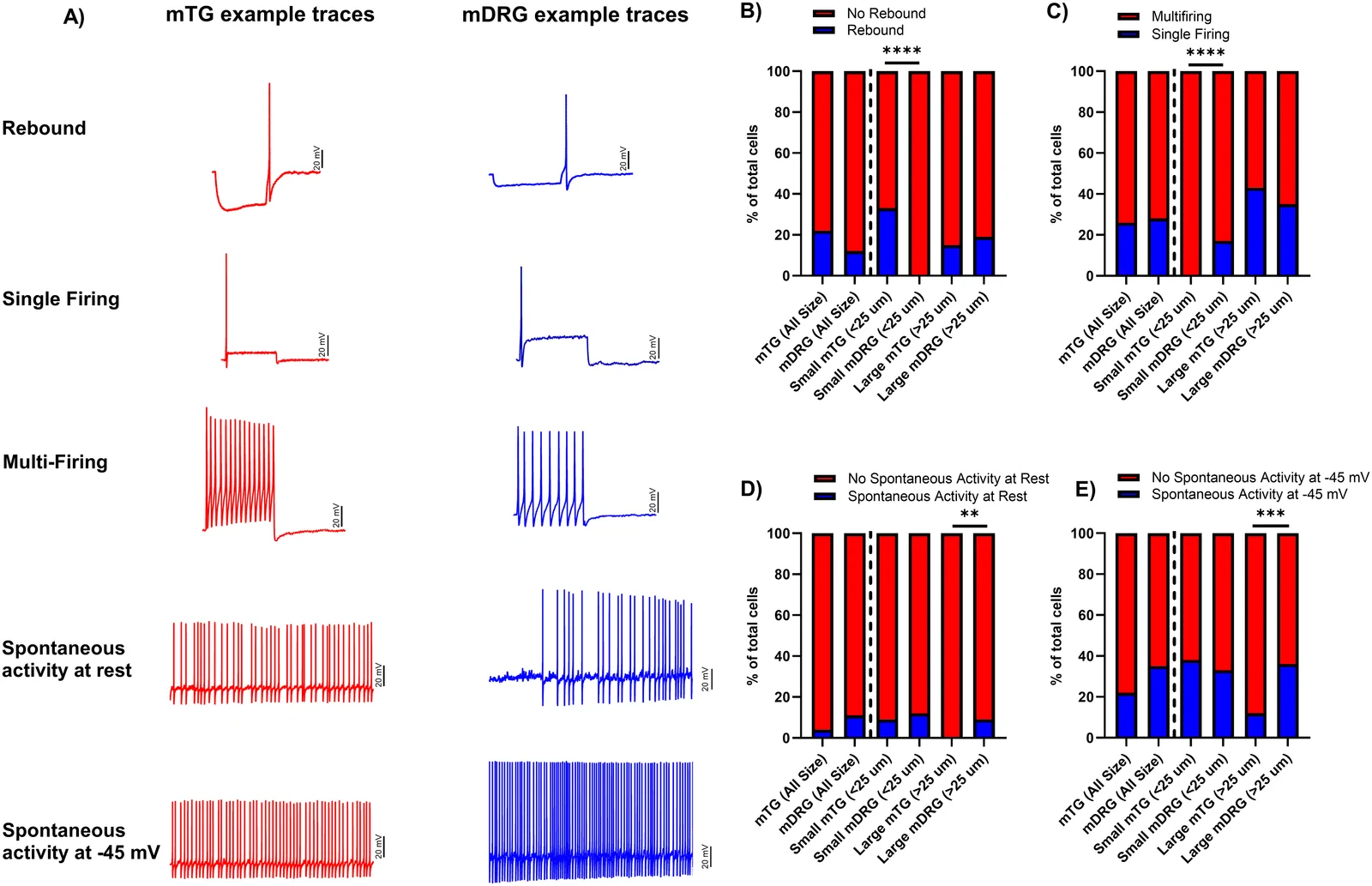
Firing phenotypes. ***A***, Example traces of phenotypes observed in mTG (*n* = 54) and mDRG (*n* = 65) percent of cells the pattern was observed including ***B***, prevalence of rebound firing; ***C***, prevalence of multifiring versus single firing; ***D***, prevalence of spontaneous activity at rest; and ***E***, prevalence of spontaneous activity at −45 mV (****p < 0.0001, ***p < 0.001, **p < 0.01; Fisher's exact test).

We next characterized the prevalence of distinct firing patterns in mTG and mDRG neurons (Fig. 3*A*). Analysis of firing phenotypes revealed several size-dependent differences between the two neuronal populations. Rebound firing was defined as the occurrence of an AP following the termination of the 500 ms current injection. The proportion of rebound firing neurons was not significantly different between mTG and mDRG neurons (22% [12/54] vs 12% [8/65], respectively; *p* = 0.0892; Fig. 3*B*). However, a significant size-dependent difference was observed, with rebound firing detected in 33% (7/21) of small mTG neurons but in none of the small mDRG neurons (0/24) *p* < 0.0001 (Fig. 3*B*). The prevalence of multifiring neurons, defined as cells generating more than one AP during the current injection, was similar between mTG and mDRG populations (74% [40/54] vs 72% [47/65], respectively; *p* = 0.8736; Fig. 3*C*). When neurons were stratified by size, all small mTG neurons exhibited a multifiring phenotype (100% [21/21]), compared with 83% (20/24) of small mDRG neurons (*p* < 0.0001). In contrast, no significant difference was observed between large mTG and large mDRG neurons (57% [19/33] vs 65% [27/41], respectively; *p* = 0.3102: Fig. 3*C*). Spontaneous activity was assessed during 30 s gap-free current-clamp recordings obtained either at rest or while holding the membrane potential at −45 mV. At rest, spontaneous firing was observed in 4% (2/54) of mTG neurons and 11% (7/65) of mDRG neurons, a difference that did not reach statistical significance (*p* = 0.1046; Fig. 3*D*). However, a significant size-dependent effect was identified among large neurons, as spontaneous activity at rest was absent in large mTG neurons (0/33) but present in 9% (4/41) of large mDRG neurons (*p* = 0.0032; Fig. 3*D*). Depolarization of neurons to −45 mV increased the prevalence of spontaneous firing in both populations. Nevertheless, the proportion of spontaneously active neurons at −45 mV was not significantly different between mTG and mDRG neurons overall (22% [12/54] vs 35% [23/65], respectively; *p* = 0.0596; Fig. 3*E*). A significant size-dependent difference was again observed among large neurons, with spontaneous firing present in 12% (4/33) of large mTG neurons compared with 36% (15/41) of large mDRG neurons (*p* = 0.0001; Fig. 3*E*).

### Differences in AP waveform characteristics were observed between mouse TG and DRG neurons

**Figure 4.**
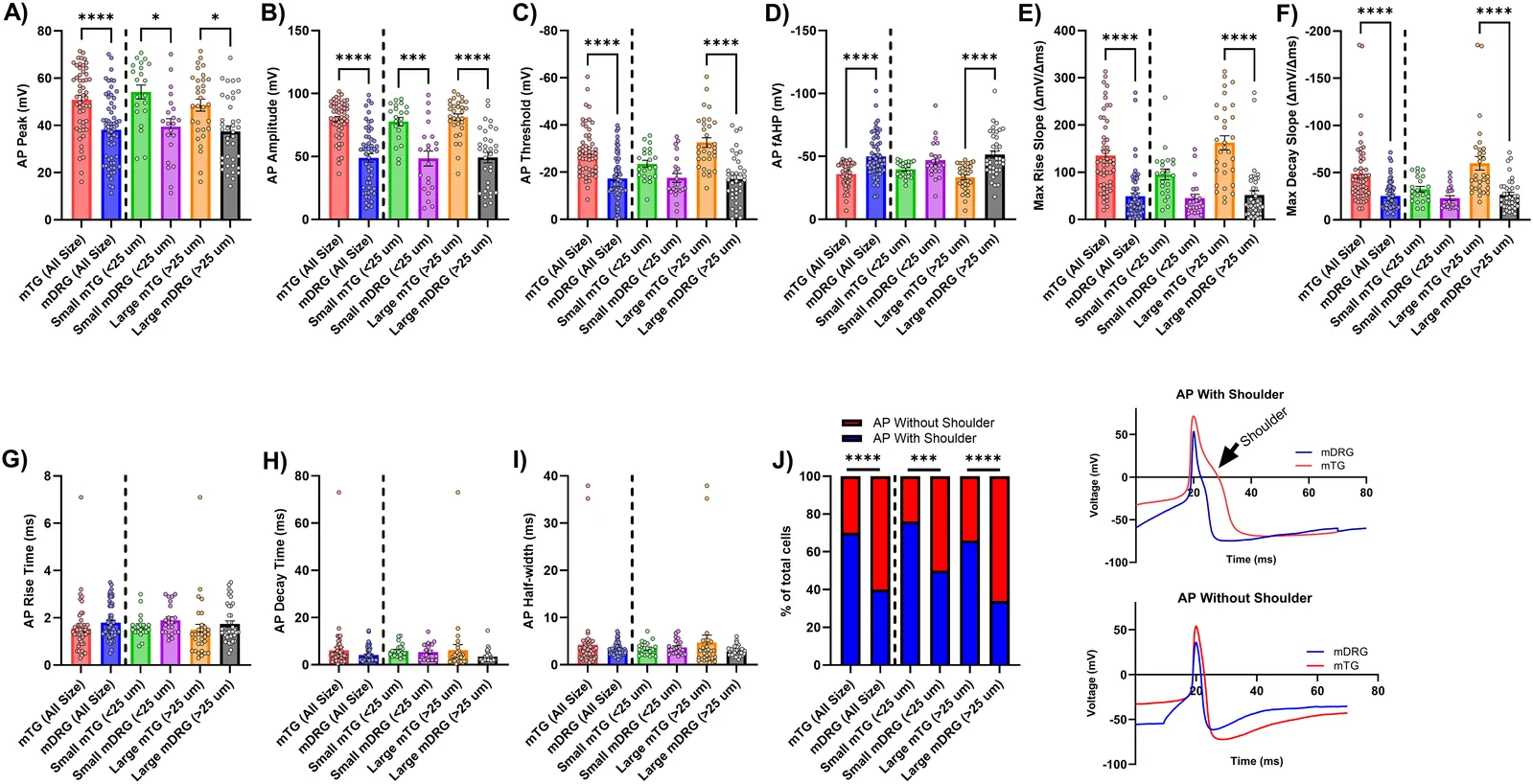
Action potential waveform characteristics. Differences in several AP waveform characteristics were observed between mTG (*n* = 54) and mDRG (*n* = 65) cells. A significant difference is found in ***A***, AP peak; ***B***, AP amplitude; ***C***, AP threshold; ***D***, AP afterhyperpolarization; ***E***, max rise slope; and ***F***, max decay slope (mTG <25 µm *n* = 21, >25 µm *n* = 33 and mDRG <25 µm *n* = 24, >25 µm *n* = 41 cells; ****p < 0.0001, ***p < 0.001, *p < 0.05; ordinary one-way ANOVA followed by Tukey's multiple comparison). No significant difference was observed in ***G***, AP rise time; ***H***, AP decay time; and ***I***, AP half-width. ***J***, Prevalence of AP shoulder (****p < 0.0001, ***p < 0.001; Fisher's exact test). Inset, Representative APs with and without shoulder. Error bars represent mean and SEM. ANOVA, analysis of variance.

AP waveform properties were compared between mTG and mDRG neurons to identify differences in intrinsic excitability. Several AP waveform parameters differed significantly between the two neuronal populations, and some of these differences were size dependent. The AP peak was significantly greater in mTG neurons than in mDRG neurons (50.84 ± 1.826 mV vs 38.14 ± 1.90 mV, respectively; *p* < 0.0001; Fig. 4*A*). This difference was observed in both small mTG versus mDRG neurons (54.15 ± 1.75 mV vs 39.51 ± 1.97 mV, respectively; *p* *=* 0.0141) and large mTG versus mDRG neurons (48.60 ± 1.84 mV vs 37.39 ± 1.89 mV, respectively; *p* *=* 0.0182; Fig. 4*A*). Similarly, AP amplitude was significantly larger in mTG neurons than in mDRG neurons (79.74 ± 1.96 mV vs 48.92 ± 3.23 mV, respectively; *p* < 0.0001; Fig. 4*B*). Significant size-dependent differences were also observed, with small mTG neurons exhibiting greater AP amplitudes than small mDRG neurons (77.68 ± 1.98 mV vs 48.38 ± 3.51 mV, respectively; *p* *=* 0.0001). Likewise, large mTG neurons had larger AP amplitudes than large mDRG neurons (81.14 ± 1.96 mV vs 49.27 ± 3.09 mV, respectively; *p* < 0.0001; Fig. 4*B*). AP threshold was significantly lower in mTG neurons compared with mDRG neurons (−28.89 ± 1.37 mV vs −7.28 ± 1.24 mV, respectively; *p* < 0.0001; Fig. 4*C*). This difference was particularly evident in large neurons, where mTG neurons exhibited a lower threshold than mDRG neurons (−32.54 ± 1.45 mV vs −17.12 ± 1.32 mV; *p* < 0.0001; Fig. 4*C*). The afterhyperpolarization (fAHP) following the AP also differed significantly between the two groups. mDRG neurons exhibited a lower fAHP than mTG neurons (−49.89 ± 2.15 mV vs −5.93 ± 1.23 mV, respectively; *p* < 0.0001; Fig. 4*D*). A significant size-dependent difference was observed among large neurons, with large mDRG neurons displaying a less pronounced fAHP than large mTG neurons (−51.49 ± 2.27 mV vs −33.38 ± 1.37 mV, respectively; *p* < 0.0001; Fig. 4*D*). AP duration was evaluated by measuring rise time, decay time, and half-width. mTG neurons exhibited a greater max rise slope than mDRG neurons (135.41 ± 10.24 mV/ms vs 49.1 ± 6.78 mV/ms, respectively; *p* < 0.0001; Fig. 4*E*). This difference was particularly evident in large neurons, where mTG neurons exhibited a greater rise slope than mDRG neurons (162.39 ± 10.79 mV/ms vs 51.42 ± 7.64 mV/ms, *p* < 0.0001; Fig. 4*E*). Similarly, mTG neurons exhibited a lower max decay slope than mDRG neurons (−48.82 ± 4.5 mV/ms vs −25.2 ± 1.95 mV/ms, respectively; *p* < 0.0001; Fig. 4*F*). This difference was particularly evident in large neurons, where mTG neurons exhibited a lower decay slope than mDRG neurons (−59.97 ± 5.19 mV/ms vs −26.42 ± 2.12 mV/ms, *p* < 0.0001; Fig. 4*F*). No significant differences were detected between mTG and mDRG neurons for rise time, decay time, and half-width, and no size-dependent differences were observed (Fig. 4*G*–*I*). APs were also classified based on the presence or absence of a shoulder during the repolarization phase. APs with shoulders exhibited a distinct hump during the middle portion of the decay phase, whereas APs without shoulders lacked this feature. The prevalence of shoulder-containing APs was significantly greater in mTG neurons than that in mDRG neurons (70% [38/54] vs 40% [26/65], respectively; *p* < 0.0001; Fig. 4*J*). This difference was observed in both small mTG versus small mDRG neurons (76% [16/21] vs 50% [12/24], respectively; *p* *=* 0.0002) and large mTG vs large mDRG neurons (66% [22/33] vs 34% [14/41], respectively; *p* < 0.0001; Fig. 4*J*). Summary of these electrophysiological features are given in Table 1.

**Table 1.** Statistical comparison between groups of comparisons

Electrophysiological properties	mTG vs mDRG (all size)	mTG vs mDRG (small)	mTG vs mDRG (large)
FSL at rheobase (ms) (Fig. 1*B*)	****	*	**
Max numbers of APs above rheobase (Fig. 2*C*)	***	***	ns
Max numbers of APs during 500 pA ramp (Fig. 2*D*)	**	*	ns
Rebound firing (Fig. 3*B*)	ns	****	ns
Multifiring (Fig. 3*C*)	ns	****	ns
Spontaneous activity at rest (Fig. 3*D*)	ns	ns	**
Spontaneous activity at −45 mV (Fig. 3*E*)	ns	ns	***
AP peak (mV) (Fig. 4*A*)	****	*	*
AP amplitude (mV) (Fig. 4*B*)	****	***	****
AP threshold (mV) (Fig. 4*C*)	****	ns	****
AP AHP (mV; Fig. 4*D*)	****	ns	****
Max rise slope (Fig. 4*E*)	****	ns	****
Max decay slope (Fig. 4*F*)	****	ns	****
AP shoulder (Fig. 4*J*)	****	***	****

Differences in several electrophysiological properties were observed between mTG and mDRG cells (****p < 0.0001, ***p < 0.001, **p < 0.01, *p < 0.05).

### Principal component analysis reveals size- and tissue-dependent differences in sensory neuron electrophysiology

**Figure 5.**
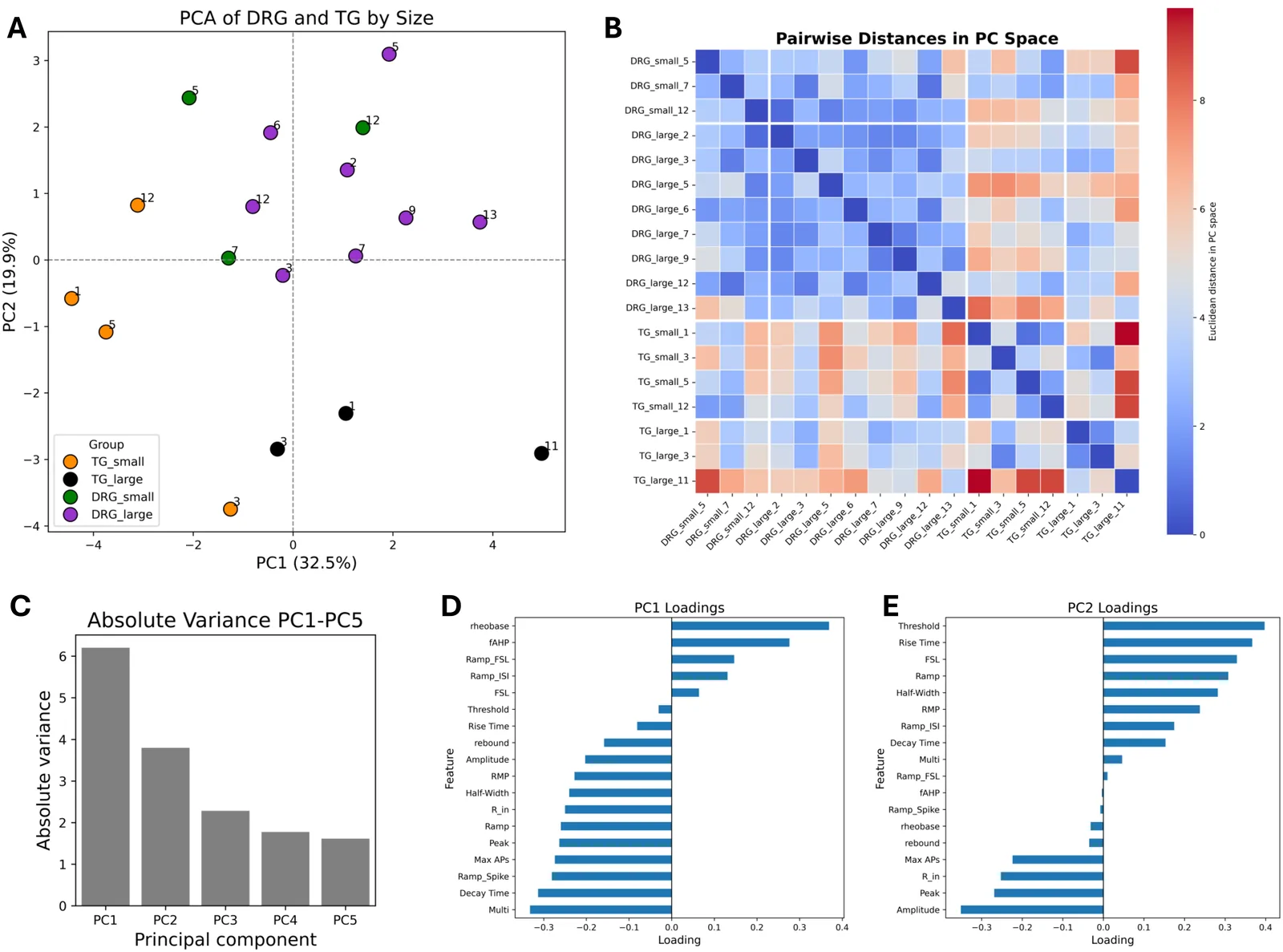
PCA reveals electrophysiological differences between TG and DRG neurons separated by soma size. ***A***, PCA of mouse-averaged electrophysiological properties from small TG (orange), large TG (black), small DRG (green), and large DRG (purple) neurons. Each point represents the average of all neurons from a single mouse within that tissue/size group. ***B***, Heatmap showing pairwise Euclidean distances in PC1–PC2 space between mouse averages, ordered by group (DRG small, DRG large, TG small, TG large). ***C***, Absolute variance explained by the first five principal components. PC1 and PC2 account for the largest proportion of variance in the dataset. ***D***, ***E***, Feature loadings for PC1 and PC2. Positive and negative values indicate the direction and relative contribution of each electrophysiological parameter to separation along the corresponding principal component.

To determine whether mouse-averaged electrophysiological properties segregated by tissue and soma size, we performed PCA on mouse-averaged datasets generated from small and large TG and DRG neurons (Fig. 5*A*). PCA revealed that DRG neurons clustered together regardless of size, whereas TG neurons were distinct from DRG and further segregated by size, indicating that both tissue identity and neuron size contribute to electrophysiological diversity. Along PC1, TG small neurons were largely separated from DRG populations, whereas large TG neurons segregated strongly along PC2, suggesting that distinct combinations of intrinsic excitability and action potential waveform features contribute to the separation of TG and DRG neurons by size class.

Pairwise Euclidean distance analysis in PC1–PC2 space further supported segregation of the four groups (Fig. 5*B*). DRG small and DRG large populations generally exhibited lower pairwise distances to one another than to TG populations, suggesting greater overall similarity between DRG neurons across size classes. In contrast, TG small neurons were more distinct from DRG groups, while large TG neurons displayed greater heterogeneity, with one TG large mouse showing relatively large distances from most other samples.

Examination of the variance explained by each principal component showed that PC1 and PC2 captured the largest proportion of total variance in the dataset, accounting for 32.5 and 19.9% of the variance, respectively, whereas subsequent components each contributed substantially less (Fig. 5*A*,*C*). To identify the electrophysiological properties contributing most strongly to group separation, we examined the feature loadings for PC1 and PC2 (Fig. 5*D*,*E*). PC1 was driven most strongly by measures related to excitability and repetitive firing, including rheobase, afterhyperpolarization, ramp first spike latency, ramp interspike interval, and first spike latency, together with negative contributions from multifiring prevalence, decay time, ramp-evoked spike number, and maximum action potential number. In contrast, PC2 was dominated by action potential waveform properties, including threshold, rise time, first spike latency, ramp response, half-width, RMP, and ramp interspike interval, with additional negative contributions from action potential amplitude, peak, and input resistance. Together, these findings indicate that electrophysiological differences between TG and DRG neurons are shaped by both soma size and tissue-specific variation in intrinsic excitability and action potential waveform properties.

### Correlation analysis identifies tissue- and size-dependent differences in electrophysiological relationships

**Figure 6.**
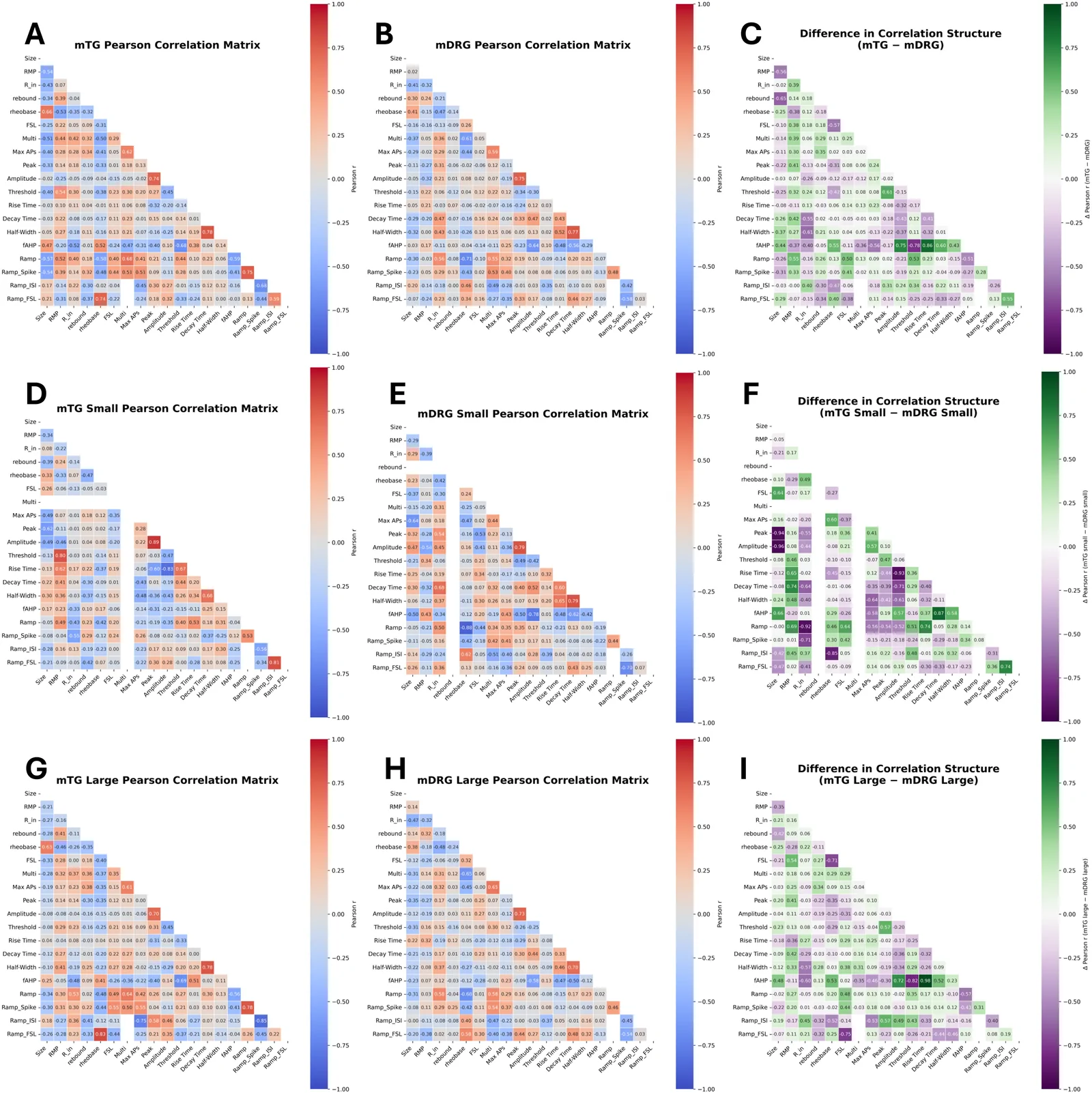
Pearson’s correlation analysis identifies coordinated electrophysiological relationships in mouse trigeminal ganglion (mTG) and dorsal root ganglion (mDRG) neurons. ***A***, ***B***, Pearson’s correlation matrices generated from all mTG and mDRG neurons, respectively. ***C***, Difference correlation matrix (mTG − mDRG) highlighting tissue-specific differences in correlation strength between electrophysiological parameters. ***D***, ***E***, Pearson’s correlation matrices for small-diameter mTG and mDRG neurons. ***F***, Difference correlation matrix (mTG Small − mDRG Small). ***G***, ***H***, Pearson’s correlation matrices for large-diameter mTG and mDRG neurons. ***I***, Difference correlation matrix (mTG Large − mDRG Large). Positive Pearson’s correlation coefficients are shown in red and negative coefficients in blue for the correlation matrices (***A***, ***B***, ***D***, ***E***, ***G***, ***H***). Difference correlation matrices (***C***, ***F***, ***I***) represent the change in Pearson’s correlation coefficients between mTG and mDRG neurons (Δ*r* = r_mTG − r_mDRG), where green indicates a more positive correlation coefficient in mTG relative to mDRG and purple indicates a more negative correlation coefficient in mTG relative to mDRG.

Pearson’s correlation analysis was performed to determine whether relationships among electrophysiological properties differed between TG and DRG neurons (Fig. 6*A*,*B*). Correlation matrices generated from all neurons revealed broadly similar overall correlation structures between tissues, with many expected relationships, such as among action potential waveform parameters, for example, AP Amplitude and AP Peak being positively correlated in both TG (*r* = 0.74) and DRG (*r* = 0.75), or among excitability measurements such as high rheobase being negatively correlated with prevalence of firing on a ramp protocol in both TG (*r* = −0.58) and DRG (*r* = −0.71). These relationships are visualized in the Pearson’s correlation (PC) plots, where each matrix displays pairwise correlations between electrophysiological features within each tissue.

To identify and quantify tissue-specific differences in electrophysiological relationships, we specifically examined how correlations between feature pairs changed between TG and DRG neurons and facilitated this comparison by generating difference correlation plots (Fig. 6*C*). These plots were created by subtracting DRG correlation coefficients from those of TG for each feature pair, enabling direct visualization of relationships that are strengthened, weakened, or reversed between tissues. Using this approach, several feature pairs were found to exhibit tissue-specific correlation patterns at the tissue level. In particular, relationships involving measures of intrinsic excitability such as rheobase, action potential threshold, and afterhyperpolarization differed between TG and DRG neurons, with these differences primarily driven by stronger or more consistent correlations in TG neurons compared with weaker or absent relationships in DRG neurons. Notably, RMP was negatively correlated with soma size in TG neurons (*r* = −0.54) but showed little relationship with soma size in DRG neurons (*r* = 0.02), indicating that membrane potential becomes increasingly hyperpolarized with increasing soma size in TG but not DRG (Δ*r* = −0.56). A prominent tissue-specific difference involved fAHP, which was substantially more integrated into the electrophysiological correlation network of TG neurons. In TG, fAHP was positively correlated with soma size (*r* = 0.47) and rheobase (*r* = 0.52), while exhibiting a negative correlation with the prevalence of multifiring neurons (*r* = −0.47). In contrast, these relationships were weak or absent in DRG neurons (size: *r* = 0.03; rheobase: *r* = −0.04; multifiring: *r* = −0.11), resulting in Δr values of 0.44, 0.55, and −0.36, respectively. These findings suggest that fAHP is more closely associated with intrinsic excitability in TG than in DRG neurons.

Small neurons exhibited substantially greater reorganization of electrophysiological relationships than was observed in the combined analysis (Fig. 6*D*–*F*). The most prominent changes involved coordination between intrinsic membrane properties and ramp-evoked excitability. In TG neurons, RMP, rebound, and first spike latency were positively correlated with prevalence of ramp firing (RMP: *r* = 0.49; rebound: *r* = 0.23; FSL: *r* = 0.20), whereas these relationships were weak or negative in DRG neurons (RMP: *r* = −0.21; rebound: no firing; FSL: *r* = −0.44), resulting in Δr values of 0.69, no value, and 0.64, respectively. In addition, several action potential waveform properties exhibited opposite relationships with soma size between tissues. Both action potential peak and amplitude were negatively correlated with soma size in TG (peak: *r* = −0.62; amplitude: *r* = −0.49) but positively correlated in DRG (peak: *r* = 0.32; amplitude: *r* = 0.47), producing some of the largest tissue-specific differences observed (Δ*r* = −0.94 and −0.96, respectively). These findings suggest that the relationships linking membrane excitability, action potential generation, and soma size differ substantially between small TG and DRG neurons.

In contrast to small neurons, tissue-specific differences in large neurons (Fig. 6*G*–*I*) were dominated by relationships involving fAHP. In TG neurons, fAHP exhibited stronger associations with multiple action potential waveform properties than in DRG neurons, including AP amplitude (Δ*r* = 0.72), AP rise time (Δ*r* = 0.98), and decay time (Δ*r* = 0.52). Conversely, the relationship between fAHP and threshold (Δ*r* = −0.82) was more negative in TG than in DRG, indicating extensive reorganization of the coupling between repolarization kinetics and action potential waveform in large sensory neurons. Additional differences were observed between rheobase and first spike latency, which were negatively correlated in TG (*r* = −0.40) but positively correlated in DRG (*r* = 0.32; Δ*r* = −0.71), suggesting tissue-specific regulation of firing threshold in large neurons.

## Discussion

Several groups have employed cultured dissociated mouse TG neurons (Guo and Cao, 2014; Avona et al., 2021; Lindquist et al., 2021; Lee et al., 2025) and mouse DRG neurons (Seitz et al., 2021; Arcas et al., 2024; Qiu and Smith, 2025) to investigate neuronal excitability using patch-clamp electrophysiology. However, to our knowledge, this is the first study to directly compare electrophysiological recordings from both cell types generated within the same laboratory, thereby minimizing potential methodological variability. In addition to conventional comparisons of intrinsic electrophysiological properties, we incorporated size-based subgroup analyses and multivariate approaches, including principal component and correlation analyses, to determine whether coordinated electrophysiological signatures distinguish TG and DRG neurons. Together, these analyses provide a more comprehensive framework for understanding the functional specialization of these two sensory ganglia.

Our findings demonstrate that mTG neurons exhibit an intrinsic electrophysiological profile consistent with enhanced stimulus-evoked excitability compared with mDRG neurons. Although common measures of excitability like RMP, rheobase, and *R*_in_ were similar between groups, mTG neurons exhibited shorter FSL, initiated repetitive firing at lower levels of current injection above rheobase, and generated a greater number of APs than mDRG-Ns. Phenotypic differences in firing patterns were also observed between the two sensory ganglia. Small mTG-Ns exhibited a greater propensity for rebound firing and repetitive (multi)firing compared with small mDRG-Ns. Together with differences in action potential waveform properties, including AP Peak, AP amplitude, AP threshold, fAHP, and max rise and decay slope, these findings suggest that the distinct excitability profiles of mTG and mDRG neurons likely reflect coordinated differences in ion channel expression and functional regulation (Jones et al., 2026; Su et al., 2026). Consistent with this interpretation, comparative transcriptomic analyses have identified differential expression of several voltage-gated potassium channels as well as voltage-gated sodium channel Na_v_1.7 between TG and DRG neurons (Megat et al., 2019). Notably, *Kcna4* (encoding K_v_1.4) and *Cacna2d1* (encoding the α2δ-1 auxiliary subunit of voltage-gated calcium channels) are enriched in DRG neurons (Megat et al., 2019), both of which have been implicated in regulating FSL (Vydyanathan et al., 2005; Duan et al., 2012; Margas et al., 2016; Zhang et al., 2023; Jones et al., 2026), providing a potential molecular explanation for the longer FSL observed in mDRG neurons. Interestingly, *Kcnq4* (encoding M-channel K_V_7.4), which contributes to fAHP and limits repetitive firing, is enriched in TG neurons (Megat et al., 2019). This finding is consistent with our multivariate correlation analyses, which demonstrated that fAHP exhibited stronger relationships with rheobase, repetitive firing, and soma size in TG than in DRG neurons, suggesting that K_v_7-dependent regulation of excitability may play a more prominent role in TG neurons (Zheng et al., 2013; King et al., 2014; Kandel et al., 2024). We also observed a higher prevalence of AP shoulders in mTG neurons, consistent with previous studies linking tetrodotoxin-resistant sodium channels (Na_v_1.8 and Na_v_1.9) to shoulder formation, repetitive firing, and enhanced excitability in nociceptive sensory neurons (Renganathan et al., 2001; Blair and Bean, 2002; Choi and Waxman, 2011; Zheng et al., 2019; Alves-Simões et al., 2025; Köster et al., 2025; Jones et al., 2026; Zhang et al., 2026). Collectively, these electrophysiological, transcriptomic, and multivariate analyses indicate that TG and DRG neurons utilize distinct mechanisms to regulate action potential initiation and repetitive firing, likely contributing to the specialized encoding of craniofacial and spinal sensory information. Further, it would be interesting to determine whether voltage-gated ion channel currents, such as Na_V_, K_V_, or Ca_V_ currents, differ between these two neuronal populations.

Small mTG neurons also exhibited a greater propensity for rebound firing than small mDRG neurons. Rebound firing has been strongly associated with T-type voltage-gated calcium channels, particularly Ca_v_3.1 and Ca_v_3.2, which promote postinhibitory depolarization and facilitate action potential initiation (Voisin et al., 2016; Weiss and Zamponi, 2019; Hoppanova and Lacinova, 2022; Timic Stamenic et al., 2025). Persistent sodium currents, particularly those mediated by Na_v_1.9, have also been implicated in promoting rebound excitability by supporting membrane depolarization following hyperpolarization (Zhou et al., 2020; Jones et al., 2026). Together, these findings suggest that enhanced rebound firing in small TG neurons may reflect coordinated regulation of T-type calcium and persistent sodium channel activity, contributing to their greater intrinsic excitability.

In contrast to the enhanced stimulus-evoked excitability observed in small TG neurons, differences in spontaneous activity were primarily identified in the large-diameter neuronal population. Spontaneous firing has been associated with persistent sodium currents mediated by Na_v_1.6, Na_v_1.7, and Na_v_1.9, as well as alterations in resting membrane conductances that lower the threshold for spontaneous depolarization (Huang et al., 2017; Xie et al., 2019; Tian et al., 2024; Vasylyev et al., 2024; Bavencoffe et al., 2025). These findings suggest that distinct molecular mechanisms underlie spontaneous and evoked excitability in TG and DRG neurons, with spontaneous activity contributing to functional specialization within large sensory neuron subpopulations.

Ultimately, the structural, functional, and sensory characteristics of peripheral neuron subtypes are largely determined by their unique gene expression profiles (Usoskin et al., 2015; Qi et al., 2024). Although TG and DRG neurons share many physiological and molecular features, several studies have demonstrated robust differences in gene expression between these sensory ganglia (Kogelman et al., 2017; Lopes et al., 2017; Megat et al., 2019; Messlinger et al., 2020; Su et al., 2026). Notably, DRG neurons express several homeobox transcription factors that are absent from the TG, whereas TG neurons exhibit enriched expression of transcripts encoding vasopressin, oxytocin, and γ-aminobutyric acid (GABA) receptor subunits (Kogelman et al., 2017; Megat et al., 2019; Messlinger et al., 2020). These transcriptional differences likely produce coordinated changes in ion channel expression and neuronal function, ultimately shaping the distinct electrophysiological phenotypes observed in each sensory ganglion. Consistent with this concept, our PCA identified distinct clustering of TG and DRG neurons based solely on electrophysiological features, paralleling the transcriptomic separation previously reported by Megat et al. (2019). Together, these findings suggest that differences in gene expression are sufficient to produce coordinated and measurable differences in sensory neuron function.

One limitation of this study is that recordings were performed exclusively in male mice. Given evidence of sex differences in the electrophysiological properties of naive DRG neurons (Patil et al., 2019; Zurek et al., 2024a), future studies should investigate whether these findings are also observed in female mice. Another limitation is that our recordings were restricted to neurons diameters mainly between 20–30 µm, which prevented organization into small-, medium-, and large-diameter neuronal groups. Nevertheless, such analyses would likely provide additional insight and should be pursued in future studies. Subtype categorization into specific neuronal subtypes using genetic reporters would also be useful.

Collectively, our findings demonstrate that TG and DRG neurons differ not only in individual electrophysiological properties but also in the coordinated organization of those properties. By integrating conventional electrophysiological comparisons with multivariate analyses, we show that ganglion-specific differences extend beyond isolated membrane characteristics to distinct electrophysiological signatures that likely reflect underlying transcriptional and molecular programs. Furthermore, our size-based analyses revealed that small- and large-diameter sensory neurons contribute differently to these functional differences, highlighting the importance of considering neuronal subtypes when investigating peripheral sensory physiology. Together, these findings provide a functional framework for understanding how excitability is differentially regulated in TG and DRG neurons and may facilitate the development of ganglion-specific therapeutic strategies for peripheral sensory diseases and disorders such as neuropathic pain.

## Synthesis

Reviewing Editor: Jibran Khokhar, Western University Department of Anatomy and Cell Biology Decisions are customarily a result of the Reviewing Editor and the peer reviewers coming together and discussing their recommendations until a consensus is reached. When revisions are invited, a fact-based synthesis statement explaining their decision and outlining what is needed to prepare a revision will be listed below. The following reviewer(s) agreed to reveal their identity: NONE. Note: If this manuscript was transferred from JNeurosci and a decision was made to accept the manuscript without peer review, a synthesis may not be available.

The authors have done a great job of addressing the reviewer comments. This is an important study with relevance to the eNeuro readership
